# Innovative technologies to manage aflatoxins in foods and feeds and the profitability of application – A review

**DOI:** 10.1016/j.foodcont.2017.01.008

**Published:** 2017-06

**Authors:** Patchimaporn Udomkun, Alexander Nimo Wiredu, Marcus Nagle, Joachim Müller, Bernard Vanlauwe, Ranajit Bandyopadhyay

**Affiliations:** a International Institute of Tropical Agriculture (IITA), Bukavu, The Democratic Republic of Congo; b International Institute of Tropical Agriculture (IITA), Nampula, Mozambique; c Universität Hohenheim, Institute of Agricultural Engineering, Tropics and Subtropics Group, Stuttgart, Germany; d International Institute of Tropical Agriculture (IITA), Nairobi, Kenya; e International Institute of Tropical Agriculture (IITA), Ibadan, Nigeria

**Keywords:** Mycotoxins, Quality control, Pre-harvest management, Post-harvest management, Technology adoption, Agricultural products

## Abstract

Aflatoxins are mainly produced by certain strains of *Aspergillus flavus*, which are found in diverse agricultural crops. In many lower-income countries, aflatoxins pose serious public health issues since the occurrence of these toxins can be considerably common and even extreme. Aflatoxins can negatively affect health of livestock and poultry due to contaminated feeds. Additionally, they significantly limit the development of international trade as a result of strict regulation in high-value markets. Due to their high stability, aflatoxins are not only a problem during cropping, but also during storage, transport, processing, and handling steps. Consequently, innovative evidence-based technologies are urgently required to minimize aflatoxin exposure. Thus far, biological control has been developed as the most innovative potential technology of controlling aflatoxin contamination in crops, which uses competitive exclusion of toxigenic strains by non-toxigenic ones. This technology is commercially applied in groundnuts maize, cottonseed, and pistachios during pre-harvest stages. Some other effective technologies such as irradiation, ozone fumigation, chemical and biological control agents, and improved packaging materials can also minimize post-harvest aflatoxins contamination in agricultural products. However, integrated adoption of these pre- and post-harvest technologies is still required for sustainable solutions to reduce aflatoxins contamination, which enhances food security, alleviates malnutrition, and strengthens economic sustainability.

## Highlights

• Evidence of adverse effect of aflatoxins on human health and trade participation.
• Highlighting recent pre- and post-harvest technologies to prevent aflatoxin contamination.
• Expressing the need for a holistic and multidisciplinary approach to manage aflatoxin contamination.
• Providing some suggestions to guide research and policies to address aflatoxin contamination.

## 1 Introduction

Food security is effectually achieved when the food pillars, including food availability, food access, food utilization, and food stability are at levels that allow all people at all times to have physical and economic access to affordable, safe, and nutritious food to meet the requirement for an active and a healthy life (FAO, 1996). When one of these four pillars weakens, then a society undermines its food security. Factors related to food insecurity and malnutrition not only influence human health and welfare, but also affect social, economic, and political aspects of society. With regards to the previous points, pre- and post-harvest losses due to mycotoxin contamination are documented as one of the driving factors of food insecurity since these substances occur along most food chains from farm to fork.

Among the different type of mycotoxins, aflatoxins (AFs) are widespread in major food crops such as maize, groundnuts, tree nuts, and dried fruits and spices as well as milk and meat products (Iqbal et al., 2015, Mutegi et al., 2009, Perrone et al., 2014). When animal feeds are infected with AF-producing fungi, AFs are introduced into animal source food chain. AFs are toxic metabolites produced via a polyketide pathway by various species and by unnamed strains of *Aspergillus* section Flavi, which includes *A. flavus*, *A. parasiticus*, *A. parvisclerotegenus*, *A. minisclerotigenes* (Pleadin et al., 2014), Strain SBG (Cotty & Cardwell, 1999), and less commonly *A. nomius* (Kurtzman, Horn, & Hessetline, 1987). Normally, *A. flavus* produces only B-type aflatoxins, whereas the other *Aspergillus* species produce both B- and G-type aflatoxins (Creppy, 2002, Zinedine and Mañes, 2009). The relative proportions and level of AF contamination depends on *Aspergillus* species, growing and storage conditions, and additional factors (Paterson & Lima, 2010). For instance, genotype, water or heat stress, soil conditions, moisture deficit, and insect infestations are influential in determining the frequency and severity of contamination (Wagacha & Muthomi, 2008). For M-type aflatoxins, these compounds are normally not found on crops, but their metabolites are found in both the meat and milk of animals whose feedstuffs have been contaminated by AF-B_1_ and AF-B_2_ (Iqbal et al., 2015, de Ruyck et al., 2015, Sherif et al., 2009).

Recently, emphasis on the health risks associated with consumption of AFs in food and feedstuffs has increased considerably. As a result of this, many experimental, clinical, and epidemiological studies have been conducted showing adverse health effects in humans and animals exposed to AFs contamination, depending on exposure (Binder et al., 2007, Fung and Clark, 2004, Sherif et al., 2009). High-dose exposure of the contaminant can result in vomiting, abdominal pain, and even possible death, while small quantities of chronic exposure may lead to liver cancer (Etzel, 2002, Sherif et al., 2009). The International Agency for Research on Cancer (IARC) has classified both B- and G-type aflatoxins as Group 1 mutagens, whereas AF-M_1_ is classified in Group 2B (IARC, 2015). Furthermore, AFs may contribute to alter and impair child growth (Turner et al., 2003, Wu and Khlangwiset, 2010). Together with other mycotoxins, AFs are commonly suspected to play a role in development of edema in malnourished people as well as in the pathogenesis of kwashiorkor in malnourished children (Coulter et al., 1986, Hendrickse, 1982). Moreover, AF contamination negatively impacts crop and animal production leading not only to natural resource waste, but also decreased market value that causes significant economic losses.

Due to these effects, different countries and some international organizations have established strict regulations in order to control AF contamination in food and feeds and also to prohibit trade of contaminated products (Juan, Ritieni & Mañes, 2012). The regulations on “acceptable health risk” usually depend on a country’s level of economic development, extent of consumption of high-risk crops, and the susceptibility to contamination of crops to be regulated (Kendra & Dyer, 2007). Indeed, the established safe limit of AFs for human consumption ranges 4–30 μg/kg. The EU has set the strictest standards, which establishes that any product for direct human consumption cannot be marketed with a concentration of AF-B_1_ and total AFs greater than 2 μg/kg and 4 μg/kg, respectively (European Commission-EC, 2007, European Commission-EC, 2010). Likewise, US regulations have specified the maximum acceptable limit for AFs at 20 μg/kg (Wu, 2006). However, if the EU aflatoxin standard is adopted worldwide, lower-income countries such as those in Asia and Sub-Saharan Africa will face both economic losses and additional costs related to meeting those standards. This situation requires alternative technologies at pre- and post-harvest levels aimed to minimize contamination of commercial foods and feeds, at least to ensure that AF levels remain below safe limits (Prietto et al., 2015).

Implementation of innovative technologies is invaluable to address the challenges related to AFs and their effects. Reduction of AF contamination through knowledge of pre- and post-harvest managements is one of the first steps towards an appropriate strategy to improve of agricultural productivity in a sustainable way. This has direct positive effects on enhancing the quality and nutritional value of foods, conserving natural resources, as well as advancing local and international trade by increasing competitiveness. It is important to identify and document available technologies that can effectively control and minimize aflatoxin contamination to sustain healthy living and socioeconomic development. There exists ample literature on tools for AF control and their benefits. Therefore, this review compiles data on innovative pre- and post-harvest technologies developed that can manage AF contamination in foods. The benefits of these technologies are also discussed in terms of food security, human health, and economic value. Finally, implications for research and management policies addressing AF issues are highlighted.

## 2 Innovative management strategies of AF reduction

A wide range of AF management options exist in literature. Depending on the “type” or mode of application, management has been classified in this review as pre-harvest stage, specifically biological control, while sorting technology, treatments with electromagnetic radiation, ozone fumigation, chemical control agents, biological control agents, and packaging material are grouped as post-harvest stage. Each of these groups of control/management options are discussed in this section.

### 2.1 Biological control

Non-aflatoxin forming strains of *A. flavus* have been used as a biological control for long-term crop protection against AF contamination under field conditions. Cotty (2006) stated that when the spore number of nontoxigenic strains in the soil is high, they will compete with other strains, both toxigenic and other atoxigenic, for the infection sites and essential nutrients needed for growth. Moreover, soil inoculation with nontoxigenic strains has a carryover effect, which protects crops from contamination during storage (Atehnkeng et al., 2014, Dorner and Cole, 2002). The ability of fungus to compete with closely related strains depends on several factors such as pH and soil type as well as the availability of nitrogen, carbon, water, and minerals (Ehrlich, 2014).

The International Institute of Tropical Agriculture (IITA) and the United States Department of Agriculture - Agriculture Research Service together with other partners have been researching in Africa on non-toxigenic biocontrol fungi that act through competitive exclusion strategy (Bandyopadhyay and Cotty, 2013, Grace et al., 2015). They have successfully developed several country-specific indigenous aflatoxin biocontrol products generically named as Aflasafe™ (www.aflasafe.com), which can be used on maize and groundnut (Bandyopadhyay & Cotty, 2013). This product is an eco-friendly innovative biocontrol technology that utilizes native non-toxigenic strains of *A. flavus* to naturally out-compete their aflatoxin-producing cousins (Atehnkeng et al., 2014). Aflasafe™ has been shown to consistently reduce aflatoxin contamination in maize and groundnut by 80–99% during crop development, post-harvest storage, and throughout the value chain in several countries across Africa (Grace et al., 2015). Aflasafe products have been registered for commercial use in Kenya, Nigeria, Senegal and Gambia (Bandyopadhyay et al., 2016), while products are under development in seven other African nations (Bandyopadhyay et al., 2016). Each Aflasafe™ product contains four unique atoxigenic strains of *A. flavus* widely distributed naturally in the country where it is to be applied (Atehnkeng et al., 2014; Bandyopadhyay et al., 2016).

Another study on biological control has been reported by Anjaiah, Thakur, and Koedam (2006) who found that inoculation of antagonistic strains of fluorescent *Pseudomonas*, *Bacillus* and *Trichoderma* spp. on peanuts resulted in significant reduction of pre-harvest seed infection by *A. flavus*. Garcia, Ramos, Sanchis, and Marin (2012) also demonstrated that the extract of *Equisetum arvense* and a mixture 1:1 of *Equisetum arvense* and *Stevia rebaudiana* is effective against growth of *A. flavus* and subsequent production of aflatoxin under pre-harvest conditions. Alaniz Zanon, Chiotta, Giaj-Merlera, Barros, and Chulze (2013) also observed 71% reduction in AF contamination in soils and in groundnuts when an AF competitive exclusion strain of *A. flavus* AFCHG2 was applied to Argentinian groundnuts. Similarly, Weaver et al. (2015) showed that non-toxigenic strains of *A. flavus* mitigated AF contaminations in maize through pre-harvest field application. Furthermore, Accinelli, Abbas, Vicari, and Shier (2014) evaluated the efficacy of a bioplastic-based formulation for controlling AFs in maize. The results showed that bio-control granules inoculated with *A. flavus* NRRL 30797 or NRRL 21882 reduced AF contaminations up to 90% in both non-Bt and Bt hybrids.

### 2.2 Sorting technology

Sorting processes seek to eliminate agricultural products with substandard quality. Normally sorting, especially for grains, can be achieved based on differentiation of physical properties such as colour, size, shape, and density as well as visible identification of fungal growth in affected crops. By rejecting damaged and discoloured samples, sorting operations reduce the presence of AFs as well as contaminating materials in food and feed (Fandohan et al., 2005). Phillips, Clement, and Park (1994) mentioned that floating and density separation could reduce AFs in stored groundnut kernels by up to 95%. In another report, Dickens and Whitaker (1975) and Zovico et al. (1999) reported that AF-contaminated groundnuts were eliminated by colour sorting processes, while fluorescence sorting was effective to reduce levels of AF contamination in pecan (Tyson & Clark, 1974) and pistachio (McClure & Farsaie, 1980) nuts. These observations were validated through a recent study, which showed that AFs contamination in pistachio nuts is more than 95% reduced by colour sorting (Shakerardekani, Karim, & Mirdamadiha, 2012).

Nonetheless, such physical methods are often laborious, inefficient, and impractical for in-line measurements. The application of computer-based image processing techniques is one of the most promising methods for large-scale screening of fungal and toxin contaminations in food and feed. Grains and other agricultural products contain various nutritional substances that are degraded by fungal growth, which in turn influence absorbance spectra of the material. For instance, Pearson, Wicklow, Maghirang, Xie, and Dowell (2001) reported that scattering and absorbance characteristics are influenced by the presence of *A. flavus* in the kernel since fungal development causes the endosperm to become powdery. Berardo et al. (2005) also showed that it was possible to quantify fungal infection and metabolites such as mycotoxins produced in maize grain by *Fusarium verticillioides* using Near Infrared Spectroscopy (NIRS). Wicklow and Pearson (2006) found that NIRS successfully identified kernels contaminated with AFs. Moreover, Fernández-Ibañez, Soldada, Martínez- Fernández, and de la Roza-Delgado (2009) highlighted NIRS technique as a fast and non-destructive tool for detecting mycotoxins such as AF-B_1_ in maize and barley at a level of 20 ppb. Nevertheless, NIRS only produces an average spectrum, which lacks in spatial information from the sample with respect to distribution of the chemical composition.

Hyperspectral imaging (HSI) is another method that can be employed to monitor both the distribution and composition of mycotoxins in contaminated food samples, especially grains. This method can produce both localized information and a complete NIR spectrum in each pixel (Manley, Williams, Nilsson, & Geladi, 2009). Yao et al. (2010) used hyperspectral imaging (HSI) techniques to estimate AF contamination in maize kernels inoculated with *A. flavus* spores. Wang et al. (2015) also demonstrated the potential HSI based in the Vis/NIR range for quantitative identification and distinction of AFs in inoculated maize kernels. Pearson et al. (2001) mentioned that the spectral reflectance ratio 735/1005 nm, which is located in the transition between Vis and NIR, can be analysed to identify highly contaminated AF corn kernels (>100 ppb) from those contaminated lower than 10 ppb. The observation was in agreement with other studies by Del Fiore et al. (2010) and Singh, Jayas, Paliwal, and White (2012). They reported that *Aspergillus* fungi in maize and wheat were detectable by analysing the HSI in 400–1000 nm or the fusion of HSI and digital images (Singh et al., 2012).

Another image based sorting technology has been proposed by Özlüoymak (2014), who reported that approximately 98% of the AFs in contaminated figs were successfully detected and separated by a UV light coupled with colour detection system. This method used the viability of bright greenish-yellow fluorescence (BGYF), which is produced by *A. flavus* via the oxidative action of peroxidases in living plant tissue (Hadavi, 2005, Lundadei et al., 2013) as an image screening technique for the classification of AF contaminated crops.

### 2.3 Treatments with electromagnetic radiation

Gamma (γ) radiation has been considered as an effective tool for preserving and maintaining quality of agricultural and food products (Herzallah et al., 2008, Jalili et al., 2010, Prado et al., 2003). Very high-energy photons generated by a gamma source such as cobalt-60 (^60^Co) are used to destroy pathogenic and spoilage microorganisms by causing direct damage to DNA in microbial cells (Markov et al., 2015). An additional effect of γ-irradiation is the interaction of energy with water molecules present in substrates or foods, producing free radicals and ions that attack the DNA of microorganisms (Da Silva Aquino, 2012). However, the efficiency of γ-irradiation depends on many factors, namely the number and type of fungal strain, radiation dose, composition of food, and air humidity (Da Silva Aquino, 2012, Jalili et al., 2012). Several studies have reported that γ-irradiation can be performed to decrease AF contamination as exhibited in Table 1.

**Table 1:** Reported applications of gamma irradiation against aflatoxin production in food products.

Product	Aflatoxin (s)	Moisture content %	Dose kGy	Temperature °C	Reduction %	Source
Black pepper	AF-B_1_	18	30	26–30	47	Jalili et al. (2012)
	AF-B_2_				39	
	AF-G_1_				47	
	AF-G_2_				40	
White pepper	AF-B_1_	18	30	26–30	51	Jalili et al. (2012)
	AF-B_2_				35	
	AF-G_1_				48	
	AF-G_2_				43	
Ground red chilies	Total AFs	12–17	6	25–28	81–91	Iqbal et al. (2013)
	AF-B_1_				92–98	
Raisins	AF-B_1_	–	10	25	65	Kanapitsas et al. (2015)
Maize	AF-B_1_	–	10	–	95	Markov et al. (2015)

The results on the potential of γ-irradiation for AF mitigation are somewhat conflicting. Some authors reported that AF content could be reduced even with a low-dose γ-irradiation. For example, Mahrous (2007) observed that using 5 kGy of γ-irradiation is sufficient to inhibit the growth of *A. flavus* and production of AF-B_1_ in soybean seeds over 60 days of storage without any noticeable changes in chemical composition. Similarly, Iqbal et al. (2013) mentioned that a dose of 6 kGy reduced total AFs and AF-B_1_ content by more than 80% in red chilies. However, some claimed that such reductions can be achieved only using high-dose γ-irradiation. Kanapitsas, Batrinou, Aravantinos, and Markaki (2015) for instance showed that the γ-irradiation at dose of 10 kGy led to an approximately 65% decrease of the initial AF-B_1_ accumulation in raisins samples inoculated by *A. parasiticus,* compared to the non-irradiated sample on the same day. The experiments done on naturally contaminated maize samples by Markov et al. (2015) also indicated that the irradiation with a 10 kGy dose can be used to reduce the amount of AF-B_1_ to an acceptable level without compromising animal and human health. Nevertheless, some authors argued that even more than 20 kGy of γ-irradiation is not effective in reducing AFs. The efficacy of γ-irradiation at high doses to decontaminate black and white peppers from AF-B_1_, AF-B_2_, AF-G_1_, and AF-G_2_ was reported by Jalili et al. (2012). They mentioned that a gamma irradiation of 30 kGy (the maximum allowable dosage as permitted by FDA) in samples at 18% moisture content was not sufficient to completely eradicate AFs.

Some reports can be found in literature about the application of ultraviolet (UV) irradiation as a non-thermal, economical technology for AF destruction in different food products. Atalla, Hassanein, El-Beih, and Youssef (2004) showed that AF-B_1_ and AF-G_1_ in wheat grain were completely eliminated after UV short wave (254 nm) and long wave (362 nm) was applied for 30 min, while AF-B_2_ was decreased by 50 and 74% when exposed to UV short wave and long wave for 120 min, respectively. A study of UV-C irradiation on groundnut, almond, and pistachio was performed by Jubeen, Bhatti, Khan, Hassan, and Shahid (2012). After treatment with UV-C at 265 nm for 15 min, all nut samples showed 100% degradation of AF-G_2_, while the complete elimination of AF-G_1_ was observed only in almond and pistachio. The level of AF-B_1_ was reduced by approximately 97% after UV-C irradiation for 45 min. García-Cela, Marin, Sanchis, Crespo-Sempere, and Ramos (2015) showed the potential of UV-A and UV-B irradiation, which can be used to reduce mycotoxin production from *A. carbonarius* and *A. parasiticus* in grape and pistachio media.

Another non-thermal technology called pulsed light (PL) has also been used in AF reduction. Normally, PL generates short, high-intensity flashes of broad-spectrum white light. The synergy between full spectra of ultraviolet, visible, and infrared light destroys both the cell wall and nucleic acid structure of microorganisms present on the surface of either food or packaging materials in a few seconds (Oms-Oliu, Aguiló-Aguayo, Martín-Belloso, & Soliva-Fortuny, 2010). Wang et al. (2016) investigated the effect of PL treatment of 0.52 J cm^−2^ pulse^−1^ on the production of AF-B_1_ and AF-B_2_ in rough rice inoculated with *A. flavus*. Application of PL treatment for 80 s reduced AF-B_1_ and AF-B_2_ in rough rice by 75 and 39%, respectively. Additionally, the mutagenic activity of AF-B_1_ and AF-B_2_ was completely eliminated by PL treatment, while the toxicity of these two aflatoxins decreased significantly.

Dielectric processes of radio frequency (RF) and microwave (MW) are additional alternative methods for controlling AFs contamination in agricultural products. Vearasilp, Thobunluepop, Thanapornpoonpong, Pawelzik, and von Hörsten (2015) used the RF to reduce AF-B_1_ in *Perilla frutescens* L. highland oil seed. They revealed that *A. niger*, *A. flavus,* and AF-B_1_ in seeds with an initial moisture content of 18% w.b. were highly inhibited by RF heat treatment at 90 °C for 7 min. For microwave application, 2.45 GHz MW was applied directly to hazelnuts contaminated with *A. parasiticus* by Basaran and Akhan (2010), who then documented MW effects on post-harvest safety and quality of the product. The results showed that MW treatment for 120 s was able to reduce fungal count of *A. parasiticus* on in-shell hazelnut without any noticeable change in the nutritional and organoleptic properties. Unlike microbial inhibition, MW treatment was not effective to decrease AFs in hazelnuts. Perez-Flores, Moreno-Martinez, and Mendez-Albores (2011) tested the effect of MW application during alkaline-cooking of AF contaminated maize. A 36% reduction of AF-B_1_ and 58% reduction of AF-B_2_ were observed after the maize was treated at 1650 W power output and 2450 MHz operating frequency for 5.5 min. In addition, the effectiveness of MW heating on the reduction of AF contamination in groundnuts and respective products was evaluated by Mobeen, Aftab, Asif, and Zuzzer (2011). Samples heated with MW up to 92 °C for 5 min resulted in a maximum AF-B_1_ reduction of 51.1–100%.

### 2.4 Ozone fumigation

Ozone, the triatomic form of oxygen (O_3_), is one of the most powerful disinfectants and sanitizing agents. It has been approved as Generally Recognized as Safe (GRAS) meaning it can be directly applied as an antimicrobial agent in the food industry. Normally, ozone can be produced by several methods such as electrical discharge in oxygen, electrolysis of water, photochemical, and radiochemical (Inan, Pala, & Doymaz, 2007). A primary attractive aspect of ozone is that, after reaching its half-life (20–50 min), decomposition products do not represent any hazard for the treated materials (Karaca and Velioglu, 2014, Kells et al., 2001). In post-harvest treatment, gaseous and aqueous ozone phases are applied to inactivate bacterial growth (Zorlugenç, Zorlugenç, Öztekin, & Evliya, 2008), prevent fungal decay (Palou, Crisosto, Smilanick, Adaskaveg, & Zoffoli, 2002), destroy pesticides and chemical residues (Hwang, Cash, & Zabik, 2001), control storage pests (Mendez, Maier, Mason, & Woloshuk, 2003), and degrade AFs (Kells et al., 2001, Young et al., 2006).

The mechanisms of ozone to inhibit microbial populations in food occur via the progressive oxidation of vital cellular components. Ozone oxidizes polyunsaturated fatty acids or sulfhydryl group and amino acids of enzymes, peptides, and proteins to shorter molecular fragments. In addition, ozone degrades the cell wall envelope of unsaturated lipids resulting in cell disruption and subsequent leakage of cellular contents (Daş, Gürakan, & Bayindirli, 2006). The mechanism of ozone on the degradation of AF-B_1_ and AF-G_1_ involves an electrophilic reaction on the C8-C9 double bond of the furan ring causing the formation of ozonide. These compounds are then rearranged into monozonide derivatives such as aldehydes, ketones, acids, and carbon dioxide (Diao et al., 2013, Inan et al., 2007). Since there is no C8-C9 double bond in the structure, AF-B_2_ and AF-G_2_ are more resistant to ozonisation than AF-B_1_ and AF-G_1_ (Agriopoulou et al., 2016, Chen et al., 2014). Even though the efficiency of ozone as a chemical detoxifier is high, a greater concentration is required to kill fungi or contaminated surfaces, while low concentration of ozone and short fumigation time is generally considered necessary in order to preserve product properties like colour, flavour, aroma, and vitamins (Chen et al., 2014, Ölmez and Akbas, 2009, Wu et al., 2006).

Ozone detoxification has been found by some studies to be useful to reduce AFs in food commodities as summarized in Table 2. Inan et al. (2007) observed that ozone treatment degraded AF-B_1_ in red peppers, while no significant variation in colour quality was found. Zorlugenç et al. (2008) investigated the effectiveness of gaseous ozone against microbial flora and AF-B_1_ content in dried figs. The results exhibited that *Escherichia coli*, mould, and AF-B_1_ were inactivated after ozone application. Using groundnut samples, de Alencar, Faroni, Soares Nde, da Silva, and Carvalho (2012) demonstrated the efficacy of the fungicidal and detoxifying effects of ozone against total AFs and AF-B_1_. In their study, ozone could control potential aflatoxin producing species, *A. flavus* and *A. parasiticus,* in groundnuts. The concentration of total AFs and AF-B_1_ was also reduced. A study conducted by Diao et al. (2013) showed that AF-B_1_ levels in groundnuts tend to decrease with ozone application, however the ozonolysis efficiency on AF-B_1_ was not further improved after 60 h. Moreover, in the sub-chronic toxicity experiment, they also found that ozone did not show any toxic effects in male and female rats. Chen et al. (2014) treated groundnut samples with ozone and observed that the detoxification rate of AFs increased. In addition, the results demonstrated that ozone application did not influence the contents of polyphenols, resveratrol, acids, and peroxide in treated samples. Luo et al. (2014) examined the effect of ozone treatment on the degradation of AF-B_1_ in maize and found that the toxicity of AF-B_1_ contaminated maize was diminished by ozone treatment.

**Table 2:** Reported applications of ozone against aflatoxin production in food products.

Product	Aflatoxin (s)	Concentration mg L^−1^	Time min	Reduction %	Source
Red pepper	AF-B_1_	33	60	80	Inan et al. (2007)
		66	60	93	
Dried figs	AF-B_1_	13.8	30	48.8	Zorlugenç et al. (2008)
			60	72.4	
			180	95.2	
Peanuts	Total AFs	21	5760	30	de Alencar et al. (2012)
	AF-B_1_			25	
	AF-B_1_	50	3600	89.4	Diao et al. (2013)
	Total AFs	6	30	65.8	Chen et al. (2014)
	AF-B_1_			65.9	
Corn	AF-B_1_	90	40	88	Luo et al. (2014)

### 2.5 Chemical control agents

A number of studies have determined the effect of synthetic and natural food additives on AF reduction in food products (Table 3). A prime example of this effect is citric acid on AF-B_1_ and AF-B_2_ degradation in extruded sorghum (Mendéz-Albores, Veles-Medina, Urbina-Álvarez, Martínez-Bustos, & Moreno-Martínez, 2009). Jalili and Jinap (2012) investigated the effect of sodium hydrosulphite (Na_2_S_2_O_4_) and pressure on the reduction of AFs in black pepper. The study reported that the application of 2% Na_2_S_2_O_4_ under high pressure resulted in a greater percentage reduction of AF-B_1_, AF-B_2_, AF-G_1_, and AF-G_2_, without damage to the outer layer of black pepper. Nevertheless, AF-B_2_ was found to be the most resistant against the applied treatment. Apart from that, it is evident that respiration from insects increases the temperature and moisture content of grains providing favourable conditions for fungal growth. For this reason, Barra, Etcheverry, and Nesci (2015) evaluated the efficacy of 2, 6-di (*t*-butyl)-p-cresol (BHT) and the entomopathogenic fungus *Purpureocillium lilacinum* on the accumulation of AF-B_1_ in stored maize. The results clearly showed that the highest reduction of AF-B_1_ in stored maize occurred with the combination of BHT and *Purpureocillium lilacinum*. In addition, the effects of organic acids during soaking process on the reduction of AFs in soybean media were studied by Lee, Her, and Lee (2015). The highest reduction rate of AF-B_1_ was obtained from tartaric acid followed by citric acid, lactic acid, and succinic acid, respectively. These acid treatments convert AF-B_1_ to β-keto acid that subsequently transforms to AF-D_1_, which has less toxicity than that of AF-B_1_ (Méndez-Albores, Arámbula-Villa, & Laorca-Piña, 2005). Zhang, Xiong, Tatsumi, Li, and Liu (2012) reported another novel technology that has been applied to inhibit AF contamination called acidic electrolyzed oxidizing water, which is an electrolyte solution prepared using an electrolysis apparatus with an ion-exchange membrane, used to decontaminate AF-B_1_ from naturally contaminated groundnut samples. The content of AF-B_1_ in groundnuts decreased about 85% after soaking in the solution. Remarkably, the nutritional content and colour of the groundnuts did not significantly change after treatment.

**Table 3:** Reported applications of chemical agents against aflatoxin production in food products.

Product	Aflatoxin(s)	Chemical agent	Reduction %	Source
Black pepper	AF-B_1_	2% Na_2_S_2_O_4_	96	Jalili and Jinap (2012)
	AF-B_2_	Pressure = 1.5 bar	77	
	AF-G_1_	Temp = 121 °C	100	
	AF-G_2_	Time = 15 s	100	
Maize	AF-B_1_	BHT + *P. lilacinum*	90	Barra et al. (2015)
Soybean	AF-B_1_	1 mol L^−1^ tartaric acid	95	Lee et al. (2015)
		Soaking time = 18 h		
Sorghum	AF-B_1_ + AF-B_2_	8 mol L^−1^ citric acid	59–89	Mendéz-Albores et al. (2009)
		MC = 200–300 g kg^−1^		
Peanuts	AF-B_1_	Acidic electrolyzed oxidizing water	85	Zhang et al. (2012)
		Ratio of liquid to solid = 5:1 (v m^−1^)		
		Temp = RT		
		Time = 15 min		
Peanuts	AF-B_1_	1 g oriental mustard flour/50 g sample	65	Hontanaya et al. (2015)
	AF-B_2_		86	
	AF-G_1_		97	
	AF-G_2_		100	
Piadina (Italian flatbread)	AF-B_1_	1 g oriental mustard flour/10 g sample	89	Saladino et al. (2016a)
	AF-B_2_		83	
	AF-G_1_		87	
	AF-G_2_		85	
Milk	AF-M_1_	1.21% calcium montmorillonite clay	68	Maki et al. (2016)
Urine (human)	AF-M_1_	3 g/day calcium montmorillonite clay, ACCS 100	44–54	Awuor et al. (2016)

To overcome the development of fungal resistance as well as residual toxicity posed by synthetic additives, the actions of some plant-based preservatives toward AF reduction have been studied in various food products. Hontanaya, Meca, Luciano, Mañes, and Font (2015) evaluated the effect of isothiocyanates, generated by enzymatic hydrolysis of glucosinolates, contained in oriental mustard flour. The findings showed that isothiocyanates reduced *A. parasiticus* growth in groundnut samples, whereas the AF-B_1_, AF-B_2_, AF-G_1_, and AF-G_2_ reduction ranged between 65 and 100%. Similar results were obtained by Saladino et al. (2016a), who reported the inhibition of AFs by isothiocyanates derived from oriental and yellow mustard flours in piadina (a typical Italian flatbread) contaminated with *A. parasiticus*. These results can be explained by the electrophilic property of isothiocyanates, which can bind to thiol and amino groups of amino acids, peptides, and proteins, forming conjugates, dithiocarbamate, and thiourea structures (Cejpek, Urban, Velišek, & Hrabcová, 1998) leading to enzyme inhibition and subsequently to cell death (Luciano & Holley, 2009). However, it is worth noting that ρ-hydroxybenzyl isothiocyanate (ρ-HBITC), which is formed in yellow mustard flour, is less stable than allyl isothiocyanate (AITC) from oriental mustard (Luciano & Holley, 2009). In substitution of common commercial preservatives, Quiles, Manyes, Luciano, Mañes, and Meca (2015) also applied active packaging devices containing allyl isothiocyanate to avoid the growth of *A. parasiticus* and AF production in fresh pizza crust after 30 days. Another study used neem leaves (*Azadirachta indica*) to inhibit the growth of AFs in wheat, maize, and rice during storage for 9 months (Sultana, Naseer, & Nigam, 2015). Due to fungicidal and anti-aflatoxigenic properties of neem leaves, the application of 20% neem powder fully inhibited all types of aflatoxins synthesis for 4 months in wheat and for 2 months in maize, whereas the inhibition of AF-B_2_, AF-G_1_, and AF-G_2_ was observed for 3 months in rice. Essential oils of different aromatic plants have been also used as food preservatives due to their antimicrobial properties. However, the antibiotic functions of essential oils are not yet clearly understood. Bluma and Etcheverry (2008) stated that the anti-aflatoxigenic activity of essential oils may be related to inhibition of ternary steps in AF biosynthesis involving lipid peroxidation and oxygenation. Komala, Ratnavathi, Vijay Kumar, and Das (2012) determined the antifungal potential use of eugenol, a compound derived from essential oils, against AF-B_1_ production in stored sorghum grain. Prakash et al. (2010) presented the efficacy of *Piper betle* L. essential oil against the AF-B_1_ production in some dried fruits, spices, and areca nut. Kohiyama et al. (2015) showed the inhibiting effect of thyme essential oil (*Thymus vulgaris*) against fungal development and AF production on *A. flavus* cultures. Likewise, Salas, Pok, Resnik, Pacin, and Munitz (2016) reported the possible utilization of flavanones (naringin, hesperidin, and neohesperidin) obtained as by-products from the citrus industry to inhibit the production of AFs from *A. flavus*.

Overall, few studies exist about chemical control of AFs in milk and dairy products. Firmin, Morgavi, Yiannikouris, and Boudra (2011) investigated the effect of a modified yeast cell wall extract on the excretion of AF-B_1_ and AF-M_1_ in faeces, urine, and milk. They observed that feed supplementation with modified extract cell walls of yeasts reduced the absorption of AF-B_1_, and decreased the concentration of AF-B_1_ and AF-M_1_ in ewe faeces. The results indicated that this organic material could be used to protect ruminants from chronic exposure to AFs present in feeds. Another study by Maki et al. (2016) examined the effect of calcium montmorillonite clay (Novasil Plus, NSP) in dairy feed on dry matter intake, milk yield, milk composition, vitamin A, riboflavin, and AF-M_1_. The calcium montmorillonite clay was found to reduce AF-M_1_ content in milk samples without affecting milk production and nutrition qualities. Similarly, Awuor et al. (2016) suggested that inclusion in the human diet of calcium silicate 100 (ACCS100), a calcium montmorillonite clay, may reduce aflatoxin bioavailability and potentially decrease the risk of aflatoxicosis in aflatoxin-prone areas such as in Kenya. These results can be explained by the fact that calcium montmorillonite clay binds tightly to AFs in the gastrointestinal tract, therefore reducing AFs bioavailability and distribution to the blood, liver, and other affected organs (Phillips, Lemke, & Grant, 2002).

### 2.6 Biological control agents at post-harvest processing stages

Physical and chemical detoxification methods have some disadvantages, such as loss of nutritional value, altered organoleptic properties, and undesirable effects in the product as well as high cost of equipment and practical difficulties making them infeasible, particularly for lower-income countries (Ahlberg, Joutsjoki, & Korhonen, 2015). However, biological methods based on competitive exclusion by non-toxigenic fungal strains have been reported as a promising approach for mitigating formation of mycotoxins and preventing their absorption into the human body (Farzaneh et al., 2012). Among various microorganisms, lactic acid bacteria (LAB) namely *Lactobacillus*, *Bifidobacterium*, *Propionibacterium*, and *Lactococcus* are reported to be active in terms of binding AF-B_1_ and AF-M_1_ (Ahlberg et al., 2015, El-Nezami and Gratz, 2011, Peltonen et al., 2001). The binding is most likely a surface phenomenon with a significant involvement of lactic acid and other metabolites such as phenolic compounds, hydroxyl fatty acids, hydrogen peroxide, reuterin, and proteinaceous compounds produced by LAB (Dalié, Deschamps, & Richard-Forget, 2010). Ahlberg et al. (2015) reported that AF binding seems to be strongly related to several factors such as LAB strain, matrix, temperature, pH, and incubation time. Elsanhoty, Ramadan, El-Gohery, Abol-Ela, and Azeke (2013) found that *Lactobacillus rhamnosus* was the best strain with the ability to bind to AF-B_1_ in contaminated wheat flour during bread-making process. Similar results were observed in yogurt cultured with 50% *Staphylococcus thermophiles* and *Lactobacillus bulgaricus* and 50% *Lactobacillus plantrium* with the greatest AF-M_1_ reduction observed at the end of storage (Elsanhoty, Salam, Ramadan, & Badr, 2014). Asurmendi, Pascual, Dalcero, and Barberis (2014) mentioned that LAB could inhibit AF-B_1_ production in brewer’s grains used as raw material for pig feed. More recently, Saladino, Luz, Manyes, Fernández-Franzón, and Meca (2016b) investigated the effect of LAB against AF development in bread with the results showing that AF content was reduced 84–100% allowing up to 4 days of additional shelf life.

Other microorganisms have also been reported to bind or degrade aflatoxins in foods and feeds. Shetty, Hald, and Jespersen (2007) tested the AF-B_1_ binding abilities of *Saccharomyces cerevisiae* strains *in vitro* in indigenous fermented foods from Ghana. The results indicated that some strains of *Saccharomyces cerevisiae* have high AF-B_1_ binding capacity. These binding properties could be useful for the selection of starter cultures to prevent high AF contamination levels in relevant fermented foods. Topcu, Bulat, Wishah, and Boyaci (2010) showed that 20–38% of AF-B_1_ was eliminated using probiotic culture of *Enterococcus faecium*. A study by Fan et al. (2013) also reported the protective effect of *Bacillus subtilis* ANSB060 on meat quality due to its ability to prevent AF residue absorption in the livers of broilers fed with naturally mouldy groundnut meal. Moreover, some bacteria such as *Rhodococcus erythropolis*, *Bacillus* sp., *Stenotrophomonas maltophilia*, *Mycobacterium fluoranthenivorans*, and *Nocardia corynebacterioides* have been found to degrade AF-B_1_ (Alberts et al., 2006, Hormisch et al., 2004). Even though, many *Bacillus* species are still avoided due to their nature of producing toxic compounds (Schallmey, Singh, & Ward, 2004). Farzaneh et al. (2012) recently showed that the non-toxic enzymes produced by *Bacillus subtilis* strain UTBSP1 can be used to reduce AF-B_1_ from contaminated substrates.

### 2.7 Packaging materials

In post-harvest management, packaging materials are frequently considered as the final step of product development in order to extend the preservation of food and feed products. During storage and distribution, food commodities can be affected by a range of environmental conditions, such as temperature and humidity as well as light and oxygen exposure. Overall, these factors have been reported to facilitate various physicochemical changes such as nutritional degradation and browning reactions with the latter causing undesirable colour changes. The interaction of these factors can also elevate the risks of fungal development and subsequent AF contamination (Giorni, Battilani, Pietri, & Magan, 2008). Many smallholder farmers in lower-income countries traditionally store agricultural products such as grains in containers typically made from wood, bamboo, thatch, or mud placed and covered with thatch or metal roofing sheets (Waliyar et al., 2015). Recently, metal or cement bins have been introduced as alternatives to traditional storage methods, but their high costs and difficulties with accessibility make adoption by small-scale farms limited (Hell & Mutegi, 2011). Hell, Cardwell, Setamou, and Poehling (2000) stated that even though polypropylene (PP) bags are currently used for grains storage, they are still contaminated by fungal and AFs especially when those reused bags contain *A. flavus* spores.

Several studies have reported the application of Purdue Improved Crop Storage (PICS) bags to mitigate fungal growth and resulting AF contamination. Williams, Baributsa, and Woloshuk (2014) indicated that the PICS bags successfully suppressed the development of *A. flavus* and resulting AF contamination in maize across the wide range of moisture contents in comparison to non-hermetic containers. These results correspond with Njoroge et al. (2014) who mentioned that grains stored in PICS bags absorbed less moisture than grains stored in woven polypropylene bags. This could be a result of PICS bag construction consisting of triple bagging hermetic technology with two inner liners made of high-density polyethylene (HDPE) and an outer layer woven PP. In addition, PICS bags reduced the oxygen influx and limited the escape of carbon dioxide, which can prevent the development of insects in stored grain (Murdock, Margam, Baoua, Balfe, & Shade, 2012). In Benin, Ghana, Burkina Faso, and Nigeria, Baoua, Amadou, Ousmane, Baributsa, and Murdock (2014) used PICS bags to store locally infested maize. Although 53% of maize had AF levels above 20 ppm, samples from PICS bags tended to have less accumulation than those from woven bags. Sudini et al. (2015) also evaluated the efficacy of PICS bags for protecting groundnuts from quality deterioration and aflatoxin contamination caused by *A. flavus* and found that there was less toxin production in PICS bags compared to cloth bags under similar conditions.

## 3 Benefits of innovative management

Many innovative management strategies that can potentially reduce AF contamination in food and feed chains have been identified by this review. These strategies have the potential to mitigate adverse effects of AF contamination on food security, public health, and economic development. An understanding of these benefits can motivate policy makers and value chain actors to explore effective ways of managing AFs during pre- and post-production processes.

### 3.1 Food security benefits

The quantity and quality of agricultural products are degraded by the presence of AFs, while the opposite is true when AF contamination is effectively prevented. The use of biocontrol methods for instance has been shown to reduce contamination up to 90% (Dorner, 2004), which potentially reduces complete loss of harvested or stored crops (Grace et al., 2015). As mentioned earlier the use of the PICS technology for grain storage can reduce AF contamination due to the controlled environment in the hermitic bags. For subsistent households, such measures can potentially increase availability of harvested food crop for family consumption (Murdock et al., 2012). Farmers can even afford to sell their excess produce and use the proceeds to purchase other food ingredients they do not produce themselves. Moreover, applications of innovative control technologies can ensure that products are safer to consume, thereby improving utilization efficiency. By reducing significant losses during storage, the control of AF can certify that the foodstuffs are available over extended periods of time, thereby ensuring consistent food availability. Effective control of AF contamination therefore has the potential to enhance food availability, food access, food utilization, and food stability.

### 3.2 Health benefits

AFs are a serious risk to public health, especially in low-income countries where most people consume relatively large quantities of susceptible crops such as maize or groundnuts. According to the estimation of the US Center for Disease Control and Prevention, about 4.5 billion people are chronically exposed to mycotoxins (Emmott, 2013). Prolonged exposure to even low levels of AF contamination in crops could lead to liver damage or cancer as well as to immune disorders (Hsu et al., 1991). In children, stunted growth and Kwashiorkor pathogenesis are caused by breast milk consumption or direct ingestion of AF-contaminated foods (Coulter et al., 1986, Hendrickse, 1982, Khlangwiset et al., 2011). Controlling AF contamination through the application of effective technologies could potentially avoid such health risks and have significant benefits (Khlangwiset & Wu, 2010) in a number of ways. First chronic diseases can be prevented to minimize pressure on the health facilities of an economy due to savings on cost of medication and treatment. People will have access to good quality food ingredients for health living and making work efficient labour force available for the economy.

### 3.3 Economic benefits

The economic benefits of AF reduction are observed through both domestic and high-value international trade markets. At domestic and regional levels, markets might not reward reduced AF in crops, but avoiding contamination could allow, in ideal cases, to increase the volume of sales, which would lead to elevated incomes as well as greater returns to investments for producers. Farmers who successfully inhibit AF contamination can also benefit from increased income due to greater product acceptance, higher market value, or access to high-value markets. In reality, there are numerous factors that have to be enhanced in order to create premium class products such as aflatoxin control, consumer awareness, marketing channels, aflatoxin testing, and stricter enforcement of production and market regulations. When such enabling conditions are met, it has been shown that aflatoxin-conscious market can pay a premium for aflatoxin safe products even in the domestic market in Africa (Bandyopadhyay et al., 2016). Moreover, the control of AF contamination could reduce costs the associated with consequent effects on humans, such as medical treatments, primarily of individuals suffering from liver cancer, as well as indirect costs such as pain and suffering, anxiety, and reduction in quality of life associated with exposure to AFs (Wu & Khlangwiset, 2010).

At the international level, many developed countries have established regulations to limit exposure to AFs. Some countries have different limits depending on the intended use, the strictest on human consumption, exports, and industrial products (FAO, 2004). Despite that stringent measures that makes phytosanitary standards seemingly more expensive, once suppliers internalize the economic costs of compliance in reality, greater economic benefits for society can be achieved. This is due to access to larger and more stable markets, and less incidence of disease. Controlling AF contamination in exportable agricultural commodities could maintain or even increase trade volumes and foreign earnings for exporting economies. Furthermore, the savings from such control measures could be channelled or invested in other economic sectors in order to generate additional income and propel growth and development.

## 4 Implications for research and policy

AFs are a critical problem for food safety in many lower-income countries where AF formation in key staple crops causes significant post-harvest losses and negative impacts on human life (Lewis et al., 2005, Mutegi et al., 2013). Currently, several innovative AF control technologies have shown potential to improve health and economic factors for farmers and other actors in commodity value chains. However, the efficacy, safety, and quality of these technologies must be verified prior to adoption. The feasibility of using biocontrol products depends not only on safety regulations in each individual country, but also on the accessibility of such biocontrol tools like Aflasafe™ to smallholder farmers. The ability to develop and maintain biocontrol strains from local resources, particularly in the production of Aflasafe™, are highly cost-effective and facilitate availability. Meanwhile, non-profit governmental or non-governmental organizations can also promote such products, which are particularly suitable for sustainable development. Bandyopadhyay and Cotty (2013) have mentioned that application of biocontrol technologies in conjunction with other AF management tools can profitably link farmers to markets, improve human and animal health, and increase food safety. However, biocontrol adoption still requires a flexible system that allows the use of biopesticides together with a favorable policy and institutional supports.

Furthermore, other techniques have been developed such as sorting technologies that offer numerous advantages including (1) rapid, real-time product information via non-destructive measurement, (2) reduction of laborious and destructive analytical methods, (3) continuous monitoring, and (4) integrating into existing processing lines for control and automation. However, investment costs are usually the main factor determining whether such technologies are adopted or not. For simplicity, development of cheap and portable diagnostics techniques that are adaptable to different field networks is imperative. In addition, future research should still be conducted in cooperation with final users to achieve full adoption potential. Despite technological advances, hand sorting may still be more suitable in lower-income countries where access to equipment is limited. The culls from sorting must be disposed in a manner that they do not enter the food chain, particularly of economically vulnerable populations. Still, with regulatory approval, irradiation and ozone fumigation could effectively reduce aflatoxin levels in crops, but these interventions are less applicable due to higher costs and safety concerns. Moreover, naturally infected grains have both internal and external colonization. While external contamination can be decontaminated, ozone cannot penetrate the internal sites of colonization and AF formation. Therefore, large ozone doses for a long time might be required for effective ozone decontamination. The application of chemical and biological control agents has been shown to reduce AF contamination both in animals and humans. Nonetheless, little information is available regarding the effective doses and frequencies as well as costs and efficacies. Generally, individual countries with their own specific cultural context, especially those with higher risks of AF, face public aversion to these technologies. Regarding storage methods, there is evidence that suggests hermetic technologies like PICS triple layer bags could be cost effective against key grain storage pests. They may also provide an improved alternative for insecticide-free, long-term storage of grains with minimal grain damage. However, these PICS bags may not be suitable and affordable for small-scale farmers over very large areas. The technology is also limited to cereals and grain crops.

Although there are many initiatives that aim to reduce AF contamination in lower-income countries, the lack of regulation enforcement, or even definition of acceptable limits, does not allow for their full development and implementation. In order to reduce AF contamination, it is necessary to have policies focused on: (1) raising awareness of public health impacts associated with AF contamination to all actors along the entire value chain, including families, farmers, consumers, processors, and traders; (2) estimating the lifespan of each technology and calculating their respective social and economic costs of diminishing the contamination risk at different intervention points; (3) reducing the harmful effects of AFs by implementing the appropriate pre- and post-harvest technologies; (4) investing in infrastructure with such capacity that allows to support further activities both in order to reduce AFs and to monitor contamination levels in different agricultural products; (5) establishing of reliable and effective low-cost testing methods to monitor AF contamination levels in rural areas; and (6) providing the required data and risk management tools for driven policy reforms, which create an effective regulatory environment to ensure domestic food safety in rural and urban areas and also facilitates trade opportunities in the region. Finally, governments need to solve the issue of how agricultural businesses can be enabled to operate profitably while complying with existing standards and limits of AF contamination.

## 5 Conclusions

This review has focused on different scientific research results regarding AF control in food and feeds at pre- and post-harvest levels. It is clear that high AF levels pose human health risks and also represent a barrier to expand trade in both domestic and international contexts. Overall, it is necessary to tackle existing global food insecurity issues by adopting and implementing cutting-edge technologies. Biocontrol technologies, in conjunction with other aflatoxin-management tools such as sorting technologies, storage, irradiation, ozone fumigation, chemical and biological control along with improved packaging materials have the potential to link farmers to markets, enhance international trade, improve health conditions of people and animals, and increase food safety and security. However, multidisciplinary and comprehensive research is still required to assess the potential benefits of these technologies. Overall, AF control interventions should be considered in order to improve food security, raise public health awareness, increase economic benefits, and reduce related costs for all actors in commodity value chains.
